# Sexual Behavior and Network Characteristics and Their Association with Bacterial Sexually Transmitted Infections among Black Men Who Have Sex with Men in the United States

**DOI:** 10.1371/journal.pone.0146025

**Published:** 2015-12-31

**Authors:** Hyman M. Scott, Risha Irvin, Leo Wilton, Hong Van Tieu, Chauncey Watson, Manya Magnus, Iris Chen, Charlotte Gaydos, Sophia A. Hussen, Sharon Mannheimer, Kenneth Mayer, Nancy A. Hessol, Susan Buchbinder

**Affiliations:** 1 Bridge HIV, San Francisco Department of Public Health, San Francisco, California, United States of America; 2 Department of Medicine, Johns Hopkins University, Baltimore, Maryland, United States of America; 3 Department of Human Development, State University of New York at Binghamton, Binghamton, New York, United States of America; 4 Faculty of Humanities, University of Johannesburg, Johannesburg, South Africa; 5 Laboratory of Infectious Disease Prevention, New York Blood Center, New York, New York, United States of America; 6 Division of Infectious Diseases, Columbia University Medical Center, New York, New York, United States of America; 7 Department of Epidemiology and Biostatistics, George Washington University, Washington, DC, United States of America; 8 Department of Pathology, John Hopkins University, Baltimore, Maryland, United States of America; 9 Hubert Department of Global Health, Rollins School of Public Health, Emory University, Atlanta, Georgia, United States of America; 10 Department of Medicine, Harlem Hospital, Columbia University, New York, New York, United States of America; 11 Department of Epidemiology, Mailman School of Public Health, Columbia University, New York, New York, United States of America; 12 Fenway Institute, Boston, Massachusetts, United States of America; 13 Department of Clinical Pharmacy, University of California San Francisco, San Francisco, California, United States of America; University of Missouri-Kansas City, UNITED STATES

## Abstract

**Background:**

Black men who have sex with men (MSM) have a high prevalence of bacterial sexually transmitted infections (STIs), and individual risk behavior does not fully explain the higher prevalence when compared with other MSM. Using the social-ecological framework, we evaluated individual, social and sexual network, and structural factors and their association with prevalent STIs among Black MSM.

**Methods:**

The HIV Prevention Trials Network 061 was a multi-site cohort study designed to determine the feasibility and acceptability of a multi-component intervention for Black MSM in six US cities. Baseline assessments included demographics, risk behavior, and social and sexual network questions collected information about the size, nature and connectedness of their sexual network. Logistic regression was used to estimate the odds of having any prevalent sexually transmitted infection (gonorrhea, chlamydia, or syphilis).

**Results:**

A total of 1,553 Black MSM were enrolled in this study. In multivariate analysis, older age (aOR = 0.57; 95% CI 0.49–0.66, p<0.001) was associated with a lower odds of having a prevalent STI. Compared with reporting one male sexual partner, having 2–3 partners (aOR = 1.74; 95% CI 1.08–2.81, p<0.024) or more than 4 partners (aOR = 2.29; 95% CI 1.43–3.66, p<0.001) was associated with prevalent STIs. Having both Black and non-Black sexual partners (aOR = 0.67; 95% CI 0.45–0.99, p = 0.042) was the only sexual network factor associated with prevalent STIs.

**Conclusions:**

Age and the number and racial composition of sexual partners were associated with prevalent STIs among Black MSM, while other sexual network factors were not. Further studies are needed to evaluate the effects of the individual, network, and structural factors on prevalent STIs among Black MSM to inform combination interventions to reduce STIs among these men.

## Introduction

Several studies have reported that Black men who have sex with men (MSM) have higher prevalence of both HIV infection and bacterial sexually transmitted infections (STIs) compared with non-Black MSM despite reporting similar or lower levels of individual sexual risk behaviors [1–3]. Individual risk behaviors may not be sufficient to explain this disparity in STI prevalence, therefore larger social and structural factors should also be evaluated [3–5].

The social-ecological conceptual model for understanding STI prevalence provides a framework to view STI prevalence at the individual (e.g., sexual risk behavior), social (e.g., social and sexual networks), and structural (i.e., racial and sexual orientation-based discrimination) levels [6–8]. Several network factors have been implicated in the higher risk of HIV infection among Black MSM. For example, studies have reported that Black MSM were more likely to have Black sexual partners than non-Black MSM [9–12]. Given the higher prevalence of HIV among Black MSM in many communities, and higher proportion of men who are unaware of their HIV infection, this combination can result in a higher risk of HIV acquisition [13]. Studies have also attributed having older sexual partners to the higher HIV risk of HIV acquisition among young Black MSM [11, 14]. Several studies have also demonstrated that sexual networks of Black MSM are highly interconnected (e.g., have high sexual network density) [15]. This interconnection may facilitate more rapid transmission of HIV infections through Black MSM sexual networks which tend to be smaller compared with other MSM [16]. While there have been several studies that examined sexual network characteristics in relation to HIV infections, very few studies have examined sexual network characteristics in the context of STIs among Black MSM [9, 10, 12, 15, 17, 18]. Bacterial STIs are more common and easily transmitted than HIV, and are likely driven by similar social-ecological factors, so have the potential to provide additional insight into factors that may drive these disparities.

The objective of this cross-sectional study was to describe the baseline participant characteristics associated with sexual network size and to determine the factors associated with prevalent STIs among Black MSM enrolled in HPTN 061 cohort study. Using the social-ecological framework, we hypothesized that individual, network, and structural factors will be independently associated with a prevalent bacterial STI.

## Materials and Methods

The HPTN 061 was a multi-site cohort study designed to determine the feasibility and acceptability of a multi-component intervention for Black MSM. The study was conducted in Atlanta, Boston, Los Angeles, New York City, San Francisco and Washington DC. The institutional review boards (IRBs) at all participating study sites reviewed and approved the study: Columbia University Medical Center IRB, Emory University IRB #2—Biomedical IRB (Committee A), Fenway Community Health IRB #1, George Washington University Medical IRB, New York Blood Center IRB, University of California, Los Angeles—South General Campus IRB, and San Francisco General Hospital Committee IRB #2 [19]. From July 2009 to October 2010, Black MSM were recruited from the community or as sexual network partners referred by index participants. Index participants were defined as participants who were: 1) HIV infected but unaware of their infection, or 2) previously diagnosed with HIV infection but not receiving HIV care and having unprotected sex with partners who are negative or of unknown HIV status, or 3) HIV-uninfected. Additional study details have been previously published [19, 20].

At the enrollment visit, eligibility was confirmed and written informed consent obtained.

Participants completed a behavioral assessment using audio computer-assisted self-interview (ACASI) technology, and an interview-administered social and sexual network questionnaire. All participants received HIV/STI risk-reduction counseling and a rapid HIV antibody test. If the rapid HIV test was reactive, HIV infection was confirmed by Western Blot testing. Participants with confirmed HIV infection were categorized as either: 1) “Prior HIV Diagnosis” if they reported a prior HIV diagnosis or were considered to be previously infected based on detection of antiretroviral drugs indicative of therapy in their enrollment sample; or 2) “New HIV Diagnosis” if they did not report a prior HIV diagnosis and did not have antiretroviral drugs detected in their enrollment sample. Those who either refused testing or did not have a sample available for confirmatory HIV testing were classified as “Missing” [20].

Urine and rectal swabs were tested by a nucleic acid amplification test (NAAT), GenProbe/Hologic, San Diego, CA for Neisseria gonorrhea and Chlamydia trachomatis and a blood specimen was collected for syphilis testing using both non-treponemal and treponemal tests. An advisory committee reviewed all syphilis results and, in consultation with clinicians at each site, determined a new syphilis infection. All participants testing positive for any infection were referred for treatment and medical and social services.

### Measures

#### Interviewer administered questions

Demographic characteristics and healthcare related questions (e.g., “In the past 6 months have you seen a healthcare provider”) were collected by an interviewer at the baseline visit. The study used ACASI to collect data on HIV testing and treatment, history of incarceration, religion and spirituality [21], and experiences with health care and health care providers.

The ACASI also collected data on sexual risk behaviors in the six months prior to enrollment, including number of male, female and transgender sex partners, number of new sex partners, HIV status and race/ethnicity of sex partners, partner type, number of receptive and insertive anal sex acts, number of sex acts that were protected by condoms and exchange of sex for money, drugs or goods.

Substance use: Questions on substance use in the 6 months prior to enrollment included use of marijuana; inhaled nitrates (poppers); smoked and powder cocaine; methamphetamine; heroin; non-prescribed Vicodin, Oxycontin, or Xanax; Viagra, Cialis, or Levitra; hallucinogens and injection drug use.

Perceived racism and sexuality discrimination: These were measured with 28 items such as the occurrence of “being treated rudely or disrespectfully” because of “my race” and “my sexuality”. The extent that the event bothered the participant was measured with a 5-point scale from “not at all” to “extremely” (Cronbach *α* = 0.95 for race; Cronbach α = 0.95 for sexuality) [22].

Internalized HIV stigma: This was measured for HIV infected and uninfected men separately. For HIV infected men, seven items were asked such as “People blame me for having HIV” and for HIV uninfected men, five items were asked such as “Society looks down on people who have HIV” with responses on a 5-point Likert scale from “disagree strongly” to “agree strongly” (Cronbach α = 0.82) [23].

#### Network questionnaire

The Social-Sexual Network (S-SN) Inventory questionnaire was interviewer-administered and collected information about the participant’s social network, demographics of social network members, and the potential of their network to provide economic assistance [24–27]. Additional questions assessed the size, nature and connectedness of their sexual network, and the overlap of their social and sexual networks.

Social Network: Several functional support network domains were assessed. The questions were designed to assess the level of functional support by determining the number of people in participant’s social support network (i.e., “Is there anybody that you get together with, spend time talking, relaxing or just hanging out with?”). Participants could list up to five support network members. After the five members had been listed, participants were asked to approximate how many other network members could provide support. The response categories were none, 1–2, 3–9, and 10 or more [28].

Sexual Network: The sexual network was delineated by a name generator: “I am now going to ask you about additional people in your sexual network. The people in your sexual network are people you have had anal or vaginal sex within the last six months. Although there may be people you only have had oral sex with in the last 6 months, we are not going to include them in this discussion. As a reminder, identify your partners by nickname or initials. We do not want anyone's actual first or last name.” [28].

After the social and sexual networks were listed, a detailed set of relationship questions was asked about each partner, including age, gender, and race/ethnicity. Age was collected as a categorical variable, and comparison to the participant age was made across these categories. Additional questions were asked about sexual partners such as HIV status, condom use, type of sex, and type of partner.

Sexual Network Density: The sexual network density represents the proportion of links between all members in a sexual network, out of all those that could exist if each person in the network were connected directly to every other. Sexual network density was assessed by the question: **“**In this section, we are going to ask whether the people in your sexual network have had sex with each other in the last year. You may not know for certain whether your sexual partners have had sex with each other in the last year, but if you think they probably have, say yes, and if you think they probably have not, say no.” [28].

### Statistical Analysis

The distribution of participant characteristics by sexual network size (dichotomized as 0–2 vs. ≥3 based upon a median of 3) were compared using contingency tables, and *p* values were calculated using chi-square or Fisher exact tests as appropriate. Social network size and sexual network density, presented as means and mean % respectively, were compared with Wilcoxon rank-sum tests. Unadjusted and adjusted logistic regression models were used to estimate the odds ratios and 95% confidence intervals for the independent variables and the risk of having any sexually transmitted infection. The final adjusted models were developed using a manual stepwise forward elimination process. Each candidate model was run separately to avoid excessive case-wise deletion of observations that had missing values on other unselected candidate predictors. Covariates with statistical significance of p<0.05 were retained in the final multivariate model. The final model fit was assessed using the Hosmer and Lemeshow Goodness of fit test. All statistical analyses were performed using SAS® software version 9.3 [29].

## Results

### Characteristics associated with sexual network size

A total of 1,553 Black MSM enrolled in this study and had baseline data available for analysis. For the sexual network size sub-analysis, 1,514 participants had baseline sexual network size data available, and the median sexual network size was 3 among enrolled participants (Table 1). Compared with men who reported a sexual network size of 0–2, men with a larger sexual network size were significantly younger, more likely to identify as Latino, have completed at least some college or more, and self-identify as homosexual or gay. Using data collected from ACASI, men with a larger sexual network were also more likely to report condomless receptive anal sex (61.0% vs 44.1%; p<0.001), condomless insertive anal sex (80.2% vs 70.7%; p<0.001), and use of poppers (16.1% vs 9.3%; p<0.001). Having a larger sexual network size was also associated with scoring higher on scales for internalized stigma (48.6% vs 41.3%, p = 0.006), perceived racism (26% vs 19.9%, p = 0.026), and perceived homophobia (22.8 vs 15.4; p = 0.002) but a lower likelihood of having a history of incarceration (56.5% vs 62.8%, p = 0.015).

**Table 1:** Individual, social, and structural characteristics associated with sexual network size among Black men who have sex with men in the HIV Prevention Trials Network 061 study.

	Sexual Network Size				
	0–2		≥3		
Variable	n (%) n = 903 (60)		n (%) n = 611 (40)	х^2^ (df)	p-value
*Individual*					
Age (years)				45.74 (4)	<0.001
18–20	53 (5.9)		51 (8.4)		
21–30	190 (21.0)		213 (34.9)		
31–40	174 (19.3)		105 (17.2)		
41–50	338 (37.4)		172 (28.2)		
>50	148 (16.4)		70 (11.5)		
Sex				0.01 (1)	0.910
Male	886 (98.1)		599 (98.0)		
Transgender	17 (1.88)		12 (2.0)		
Ethnicity				14.34 (1)	<0.001
Latino	51 (5.7)		67 (11.0)		
Not Latino	852 (94.4)		544 (89.0)		
Education				16.21 (3)	0.001
Less than high school degree	175 (19.4)		85 (13.9)		
HS/GED/Vocational/Trade/Tech	353 (39.1)		211 (34.6)		
Some College/2 year Degree	270 (29.9)		224 (36.7)		
4 year college degree or more	105 (11.6)		90 (14.8)		
Ever Incarcerated	555 (62.8)		342 (56.5)	5.87 (1)	0.015
Employed				0.37 (1)	0.542
Yes	276 (30.6)		196 (32.1)		
No	626 (69.4)		415 (67.9)		
Annual Household income				6.00 (4)	0.200
<10,000	352 (39.3)		213 (35.3)		
10,000–29,999	314 (35.1)		212 (35.1)		
30,000–49,999	131 (14.6)		105 (17.4)		
50,000–69,999	56 (6.3)		34 (5.6)		
≥70,000	42 (4.69)		40 (6.62)		
Has healthcare coverage				1.02 (1)	0.311
Yes	554 (61.4)		359 (58.8)		
No	349 (38.7)		252 (41.2)		
Visit to health care provider*				0.41 (1)	0.521
Yes	544 (60.2)		358 (58.6)		
No	359 (39.8)		253 (41.4)		
HIV testing, previous year	654 (73.6)		453 (74.5)	0.17 (1)	0.684
Enrollment City				9.89 (5)	0.078
New York	166 (18.4)		136 (22.3)		
Boston	154 (17.1)		76 (12.4)		
Los Angeles	157 (17.4)		121 (19.8)		
Washington DC	129 (14.3)		87 (14.2)		
Atlanta	170 (18.8)		116 (19.0)		
San Francisco	127 (14.1)		75 (12.3)		
Sexual Orientation				16.26 (2)	<0.001
Homosexual/Gay	239 (26.9)		208 (34.6)		
Bisexual	284 (32.0)		141 (23.5)		
Other	364 (41.0)		252 (41.9)		
Has a primary partner	112 (12.4)		59 (9.7)	2.77 (1)	0.096
Any receptive condomless anal sex*	393 (44.1)		370 (61.0)	41.00 (1)	<0.001
Any insertive condomless anal sex*	633 (70.7)		486 (80.2)	17.08 (1)	<0.001
Poppers	78 (9.3)		92 (16.1)	14.91(1)	<0.001
*Social and Sexual Network*					
Social Network Size (mean)	4.1		5.6		<0.001
Partner Racial composition				132.4 (2)	<0.001
Exclusively Black	582 (64.7)		263 (43.2)		
Exclusively Non-Black	142 (15.8)		58 (9.5)		
Both Black and Non-Black	175 (19.5)		288 (47.3)		
Sexual partner 2 or more age categories different	317 (35.1)		377 (61.7)	103.84 (1)	<0.001
Any condomless sex with sexual network partners	821 (91.5)		584 (95.6)	9.38 (1)	0.002
Sexual Network Density (mean %)	4.0		5.3		<0.001
Any Sexual and Social Network Partner Overlap	771 (85.4)		549 (89.9)	6.52 (1)	0.011
*Structural*					
Internal stigma				7.58 (1)	0.006
Low	509 (58.7)		306 (51.4)		
High	358 (41.3)		289 (48.6)		
Perceived racism				7.30 (2)	0.026
Never or low	246 (30.9)		154 (27.7)		
Medium	391 (49.2)		258 (46.3)		
High	158 (19.9)		145 (26.0)		
Perceived Homophobia				12.97 (2)	0.002
Never or low	335 (44.1)		197 (37.1)		
Medium	308 (40.5)		213 (40.1)		
High	117 (15.4)		121 (22.8)		
*HIV and Bacterial Sexually Transmitted Infections*					
HIV Status (Confirmed)				5.85 (3)	0.119
HIV negative		685 (75.9)	461 (75.6)		
Prior HIV diagnosis		155 (17.2)	88 (14.4)		
New HIV diagnosis		45 (5.0)	45 (7.4)		
Missing**		18 (2.0)	16 (2.6)		
Any Bacterial Sexually Transmitted Infection		127 (14.2)	126 (20.7)	10.9 (1)	<0.001
Syphilis		63 (7.2)	55 (9.2)	2.00 (1)	0.158
Urethral Gonorrhea		7 (0.8)	11 (1.82)	3.24 (1)	0.072
Rectal Gonorrhea		21 (2.5)	33 (5.7)	11.25 (2)	0.004
Urethral Chlamydia		15 (1.7)	15 (2.5)	1.16 (1)	0.282
Rectal Chlamydia		47 (5.5)	51 (8.8)	5.67 (1)	0.017

* In the past six months.

** Participants who refused HIV testing or without samples available for confirmatory testing.

Using responses from the SSN Inventory, men with a larger sexual network reported a larger mean social network size (5.6 vs. 4.1, p<0.001). Men with a larger sexual network were less likely to report exclusively Black sexual partners (43.2% vs. 64.7%; p<0.001); they were more likely to have sexual partners at least two age categories different (61.7% vs. 35.1%; p<0.001), higher sexual network density (5.3% vs. 4.0%; p <0.001), and overlap in their social and sexual network (89% vs. 85%; p<0.011). Having a larger sexual network size was also associated with a higher odds of condomless receptive anal sex (61.0% vs. 44.1%, p = <0.001) and condomless insertive anal sex (80.2% vs. 70.7%, p<0.001) with sexual network partners.

There were 253 participants with one or more bacterial STIs diagnosed at baseline in this study. Reporting a larger sexual network size was associated with a higher risk of having rectal gonorrhea (5.7% vs 2.5%; p = 0.004) and rectal chlamydia (8.8% vs 5.5%) (Table 1) compared with reporting a smaller sexual network size. Confirmed HIV status was not associated with sexual network size.

### Factors associated with the odds of having a prevalent STI

In the adjusted logistic regression models, older age (aOR = 0.57; 95% CI 0.49–0.66, p<0.001), and having both Black and non-Black sexual partners (aOR = 0.67; 95% CI 0.45–0.99, p = 0.042) were associated with a lower odds of having a prevalent STI (Table 2). Compared with reporting one male sexual partner, having 2–3 partners (aOR = 1.74; 95% CI 1.08–2.81, p<0.024), or more than 4 partners (aOR = 2.29; 95% CI 1.43–3.66, p<0.001) was associated with having a prevalent STI. Men with a confirmed prior HIV diagnosis (aOR = 3.21; 95% CI 2.13–4.82, p<0.001), new HIV diagnosis (aOR = 2.55; 95% CI 1.51–4.30, p<0.001), or missing a confirmed HIV status (aOR = 2.63; 95% CI 1.06–6.55, p<0.037) had higher odds of a prevalent STI compared with men who were confirmed HIV negative. Popper use was the only substance associated with prevalent STIs in the adjusted analysis (aOR = 1.58; 95%CI 1.03–2.43, p<0.037). Other substances, included heavy alcohol use, were not found to be associated with prevalent STIs so were not included in the model (data not shown). Site of enrollment was also associated with prevalent STIs: compared to the New York site, the Washington DC site had the highest odds (aOR = 2.34; 95%CI 1.46–3.75, p<0.001) and the Boston site had the lowest odds (aOR = 0.34; 95%CI 0.16–0.71, p = 0.004). Although having a larger sexual network size was associated with an increased odds of having a prevalent STI in unadjusted analysis, this association was no longer statistically significant in the adjusted analysis. Furthermore, there was no significant association between prevalent STIs and other sexual network variables–sexual network density, overlap of sexual and social network, or having sexual network partners who were more than two age categories different–in either unadjusted or adjusted analysis. The adjusted model fit the data well (Goodness of Fit chi-square p-value = 0.37).

**Table 2:** Unadjusted and adjusted logistic regression for the odds of having a prevalent bacterial sexually transmitted infection among Black men who have sex with men in the HIV Prevention Trials Network 061 study.

	Univariate		Multivariate	
Variable	Any STI OR (95% CI)	p value	Any STI OR (95% CI)	p value
*Individual*				
Age (per decade)	0.59 (0.52–0.67)	<0.001	0.57 (0.49–0.66)	<0.001
Education				
Less than high school degree	0.86 (0.57–1.31)	0.486		
HS/GED/Vocational/Trade/Tech	1			
Some College/2 year Degree	1.25 (0.91–1.71)	0.172		
4 year college degree or more	1.26 (0.83–1.92)	0.273		
Ever Incarcerated	0.73 (0.56–0.96)	0.025		
Annual Household income				
<10,000	0.46 (0.28–0.77)	0.003		
10,000–29,999	0.56 (0.34–0.94)	0.026		
30,000–49,999	0.86 (0.50–1.48)	0.586		
50,000–69,999	0.69 (0.34–1.38)	0.295		
>70,000	1			
HIV Status (Confirmed)*				
HIV negative	1		1	-
Prior HIV diagnosis	2.08 (1.49–2.90)	<0.001	3.21 (2.13–4.82)	<0.001
New HIV diagnosis	2.96 (1.86–4.71)	<0.001	2.55 (1.51–4.30)	<0.001
Missing	3.05 (1.41–6.61)	0.005	2.63 (1.06–6.55)	0.037
Enrollment Site				
New York	1		1	-
Boston	0.27 (0.14–0.54)	<0.001	0.34 (0.16–0.71)	0.004
Los Angeles	1.28 (0.83–1.97)	0.269	1.29 (0.80–2.07)	0.300
Washington DC	2.83 (1.86–4.30)	<0.001	2.34 (1.46–3.75)	<0.001
Atlanta	1.37 (0.89–2.10)	0.152	1.51 (0.93–2.46)	0.097
San Francisco	0.48 (0.26–0.88)	0.017	0.77 (0.40–1.47)	0.423
Number of Male Sex Partners				
1	1		1	-
2–3	1.71 (1.11–2.63)	0.145	1.74 (1.08–2.81)	0.024
>4	2.56 (1.70–3.85)	<0.001	2.29 (1.43–3.66)	<0.001
Receptive condomless anal sex	1.91 (1.45–2.52)	<0.001		
Insertive condomless anal sex	0.75 (0.56–1.01)	<0.001		
Poppers**	1.75 (1.20–2.55)	<0.001	1.58 (1.03–2.43)	0.037
Has healthcare coverage	0.69 (0.53–0.91)	0.008		
Visit to healthcare provider**	0.75 (0.57–0.98)	0.035		
Unemployed	0.65 (0.49–0.85)	0.002		
Gay or Homosexual Identity	2.17 (1.64–2.88)	<0.001		
HIV Test in previous 12 months	0.86 (0.64–1.16)	0.316		
Has a primary partner	0.80 (0.51–1.25)	0.323		
*Social and Sexual Network*				
Social Network Size	1.03 (0.97–1.09)	0.322		
Sexual Network Size				
0–2	1			
>3	1.58 (1.20–2.07)	0.001		
Partner Racial composition				
Exclusively Black	1		1	-
Exclusively Non-Black	0.68 (0.44–1.05)	0.080	0.89 (0.54–1.48)	0.657
Both Black and Non-Black	0.61 (0.45–0.84)	0.003	0.67 (0.45–0.99)	0.042
Sexual partner 2 or more age categories different				
Yes	0.87 (0.66–1.14)	0.317		
No	1			
Any condomless anal sex with sexual network partners.				
Yes	1.43 (0.78–2.60)	0.240		
No	1			
Any Sexual and Social Network Partner Overlap				
Yes	1.17 (0.78–1.74)	0.458		
No	1			
Sexual Network Density^a^	0.93 (0.84–1.03)	0.160		
*Structural*				
Internal Stigma	1.04 (1.01–1.07)	0.014		
Perceived Racism				
Low	1			
Medium	1.06 (0.77–1.46)	0.716		
High	0.61 (0.40–0.94)	0.024		
Perceived Homophobia				
Low	1			
Medium	1.06 (0.78–1.46)	0.702		
High	1.08 (0.72–1.61)	0.714		

* Participants who have missing HIV status confirmation includes subjects who refused testing and those with missing confirmatory samples.

**Prior six months.

^a^ per 10% increase in density

## Discussion

We observed that sexual partner racial composition was the only sexual network characteristic independently associated with having a prevalent STI among this large cohort of Black MSM. A previously reported national probability sample of men and women in the US observed that Black men and women were more likely than non-Black men and women to have disassortative mixing by STI risk and assortative mixing by race [30]. This mixing pattern, coupled with a smaller sexual networks, has been proposed as a potential explanation for the racial/ethnic STI/HIV disparities observed in the U.S [16]. Although, similar sexual network mixing patterns have also been described among Black MSM, these analyses have primarily focused on HIV risk [9, 10, 31]. Furthermore, our analysis adjusted for a combination of social and structural factors using the social-ecological framework thus providing additional support for the role of racially assortative mixing and the prevalence of STIs. A better understanding of sexual networks and associated risk factors has important implications for potentially utilizing networks as part of STI prevention interventions.

This investigation also identified that younger age and homosexual or gay-identity were associated with having a larger sexual network size among Black MSM. The larger sexual networks of younger Black MSM in this cohort may facilitate other network factors associated with increased risk of HIV and STIs, such as bridging of behaviorally high and low risk network members, sexual partner concurrency, and having a sexual partner with an STI [30, 32–34]. Consistent with prior reports that Black MSM are less likely than other MSM to identify as gay, less than one third of the Black MSM in this cohort self-identified as gay with the largest proportion identifying as “Other” [35]. Several studies have reported that gay-identified Black MSM are more likely to report higher sexual risk behavior, such as condomless anal sex and a higher number of sexual partners, compared with non-gay identified Black MSM [36–38]. The association between gay identity and sexual risk may be mediated by social factors of gay community involvement and associated sexual risk norms [36, 39]. Understanding how to best leverage the lower reported risk among non-gay identified Black MSM as a resiliency factor may be a potential component of a multi-level HIV and STI prevention strategy.

We did not identify other sexual network characteristics, such as sexual network size or density, as significant predictors of prevalent STIs among this cohort of Black MSM. While previous studies have identified these sexual network factors as potential explanations for the disparity in HIV infection among Black MSM compared with other MSM, they were not significant predictors of prevalent STIs among this large cohort of Black MSM [2, 12, 15, 18, 34]. Sexual network size was the only sexual network variable significant in the unadjusted analysis, but not in the adjusted analysis. One potential explanation may be the adjustment for individual sexual risk behavior (i.e., number of sexual partners), as assessed through ACASI, in our adjusted model. For example, participants may have reported additional sexual partners who are not identified as part of the sexual network (e.g., anonymous sexual partners), but contribute to STI risk. Furthermore, Smith and colleagues found that network factors were strongly linked to individual sexual behavior, emphasizing the important influence of social norms in individual risk behavior [40]. Although their analysis was focused on social networks, it does suggest the importance of looking at individual and network-level characteristics together.

In the adjusted hierarchal social-ecological model, we found that perceived racism, as measured in this study, was not associated with prevalent STIs. Most prior studies analyzing the association of structural discrimination and STIs have focused on STI testing and not STI prevalence [41]. Ford et al. reported that perceived everyday racism was not associated with lower uptake of HIV testing among STI clinic attendees [42]. Irvin et. al., using data from this cohort, also reported that experiences of racism were not associated with lower healthcare utilization and HIV testing among Black MSM [43]. These studies suggest that structural discrimination (as measured in these studies) may not fully explain the disparity in HIV and STIs, and are consistent with the finding from other studies that perceived racial discrimination is not associated with lower utilization of many preventative care services [44–46]. However, prior work by Ayala et al. demonstrates a positive association with experiences of racism and discordant condomless anal sex among Black MSM, mediated by a pathway through sexual situations which may make condom use more difficult (i.e., having sex in a casual partner’s home) [47].

Given that the vast majority of STIs among MSM are asymptomatic, utilization of regular STI screening is an important strategy to identify and treat infections and thus prevent further transmission [48]. Secondary to many structural barriers including higher unemployment rates, Black MSM are less likely than other racial/ethnic groups to have health insurance [3, 49]. A recent study by Sullivan et al., showed that the large racial disparity in HIV incidence between Black and White MSM was mediated by healthcare status and partner race [50]. We found that partner race was associated with prevalent STIs among this large cohort of Black MSM, but healthcare status was not found to be a significant predictor in our adjusted analysis. Further investigation is needed into other structural barriers that may be positively associated with prevalent STIs among Black MSM.

Confirmed HIV status was associated with prevalent STIs in this study, but not with sexual network size. In the adjusted analysis, men with a previous or new HIV diagnosis had higher risk of a prevalent STI compared with HIV-negative men. These findings are consistent with previous reports of elevated STI risk among HIV-positive MSM. In a recent cross-sectional analysis, Turner et al. reported that HIV positive MSM had a more than two-fold risk of rectal chlamydia, but no increased risk for rectal gonorrhea [51]. Similarly, annual primary and secondary syphilis cases have increased among MSM, and data from San Francisco showed an increased risk among HIV positive MSM [52, 53]. Seroadaptive behavior has been proposed as a possible explanation for increased rates of STIs among MSM overall; however, there have been few published reports of increased STIs among HIV-positive MSM compared with HIV-negative MSM [54]. The increased STI risk among HIV-positive MSM, even after adjustment for sexual risk behavior, warrant further exploration for additional sexual network factors that may drive the increased risk of prevalent STIs.

This study had several limitations worth noting. The study population was a community-recruited cohort of Black MSM but sites were able to use a variety of recruitment methods and venues. The differences in recruitment methods between sites (i.e., some sites recruited more heavily at STI clinics than others) may have introduced selection bias into our sample. This may have led to differences between the sites, and the sample being less representative of community-dwelling Black MSM in the US. Sites that recruited from STI clinics may also have differential recruitment of Black MSM not only with STIs themselves but also with partners who have STIs, distorting the distribution of both predictors and outcomes of interest. However, we adjusted for enrollment site in our multivariate analysis to take these important differences into account. Furthermore, while we included variables from multiple levels (e.g., individual, social, and structural) within the social-ecological conceptual model, not all variables were measured in the context of this study. This study included individual sexual risk behavior in the adjusted model for factors associated with prevalent STIs, but not all possible individual-level factors (e.g., psychosocial factors). Furthermore, our stigma measures were limited to internal stigma, and did not capture all the social and structural domains of stigma. In addition, the sexual network data is subject to social desirability bias and recall bias. While ACASI was used to obtain behavioral risk data and may have reduced social desirability bias, the sexual network data were obtained through a structured in-person interviewer. This may have led to under-reporting of sexual network partners and the interconnectedness that was used to determine sexual network density. The sexual network density was collected from an ego-centric perspective, so it is also likely that study participants did not have full knowledge about the connections between their sexual network partners. This would likely result in an underestimation of the calculated sexual network density. Finally, although this was the largest cohort study of Black MSM to date, the sample size was insufficient to evaluate the association with sexual network size and individual bacterial STIs.

## Conclusions

We found prevalent bacterial STIs and several demographic and structural factors, including perceived racism, were associated with reporting larger sexual network size among Black MSM. However, sexual network size does not appear to be as important as the number and racial composition of sexual partners in predicting prevalent STI when adjusting for other factors using the social-ecological model. Further studies are needed to evaluate the effects of the individual, partner, social, and structural factors on both prevalent and incident STIs among Black MSM. Understanding these effects will be needed to appropriate design and target interventions to reduce STIs among these men.
